# The integrated transcriptome bioinformatics analysis identifies key genes and cellular components for proliferative diabetic retinopathy

**DOI:** 10.1371/journal.pone.0277952

**Published:** 2022-11-21

**Authors:** Nan Gao, Shengli Hao, Guannan Huang, Weiting Hao, Long Su

**Affiliations:** Department of Ophthalmology, The Second Hospital of Tianjin Medical University, Tianjin, China; Indiana University Purdue University at Indianapolis, UNITED STATES

## Abstract

Proliferative Diabetic Retinopathy (PDR) is a chronic complication of Diabetes and the main cause of blindness among the world’s working population at present. While there have been many studies on the pathogenesis of PDR, its intrinsic molecular mechanisms have not yet been fully elucidated. In recent years, several studies have employed bulk RNA-sequencing (RNA-seq) and single-cell RNA sequencing (scRNA-seq) to profile differentially expressed genes (DEGs) and cellular components associated with PDR. This study adds to this expanding body of work by identifying PDR’s target genes and cellular components by conducting an integrated transcriptome bioinformatics analysis. This study integrately examined two public bulk RNA-seq datasets(including 11 PDR patients and 7 controls) and one single-cell RNA-seq datasets(including 5 PDR patients) of Fibro (Vascular) Membranes (FVMs) from PDR patients and control. A total of 176 genes were identified as DEGs between PDR patients and control among both bulk RNA-seq datasets. Based on these DEGs, 14 proteins were identified in the protein overlap within the significant ligand-receptor interactions of retinal FVMs and Protein-Protein Interaction (PPI) network, three of which were associated with PDR (CD44, ICAM1, POSTN), and POSTN might act as key ligand. This finding may provide novel gene signatures and therapeutic targets for PDR.

## Introduction

Proliferative Diabetic Retinopathy (PDR) is a slow-onset, chronic complication of diabetes and the main cause of blindness among the world’s working population at present [1]. The global number of diabetic patients is expected to rise to 366 million by 2030, with 11% of them developing vision-threatening retinopathy [2, 3], making early diagnosis and treatment of PDR critical. While studies have previously examined the roles of inflammation, oxidative stress and cytokine production/release in PDR’s pathogenesis [3], its underlying molecular mechanism has not been fully elucidated, and more studies are needed to provide a deeper understanding for uncovering more effective therapeutic targets.

The identification of specific gene expression patterns can help understand disease pathogenesis and reveal possible theraputeic targets [4]. Recently, bulk RNA-sequencing (RNA-seq) and single-cell RNA sequencing (scRNA-seq) have been widely used for gene expression profiling and identifying cell populations in Fibro (Vascular) Membranes (FVMs) from PDR patients [5]. Still, to the best of our knowledge, no previous study on PDR has integrated RNA-seq and scRNA-seq to identify Differentially Expressed Genes (DEGs) between normal FVMs and that from PDR patients. Therefore, this study conducts an integrated transcriptomics analysis through RNA-seq and scRNA-seq. We identified three biomarkers of PDR, among which POSTN and FAK/Akt pathway may be potential therapeutic targets of PDR.

## Data and methods

### Data collection

The gene expression datasets of PDR were obtained through the Gene Expression Omnibus (GEO) database (http://www.ncbi.nlm.nih.gov/) [6]. Two bulk RNA-seq datasets (GSE94019 and GSE102485, containing SRR5925083-SRR5925086, SRR5925099- SRR5925100) [7, 8] were downloaded to profile DEGs, including FVMs from 11 PDR patients and seven controls. A single-cell RNA-seq dataset consisting of 7,971 FVM cells from five PDR patients was also downloaded from the GEO database (GSE165784) [5]. Validation studies were then conducted using data from a microarray dataset (GSE60436) comprised of six PDR patients and three control subjects [9].

### Processing of RNA-seq data

SRA Toolkit (version 2.11.3-ubuntu64) was used to download and preprocess the raw data from the two datasets. Raw reads were first separated into FASTQ files of pair-end reads, with FastQC (version 0.11.5) used for data quality control. The clean reads were aligned to the human reference genome (USCS hg19) by HISAT2 (version 2.1.0). Finally, SAMtools (version 1.9) and HTSeq (version 0.6.1p1) were used to quantify and map the reads to an annotated document (GENCODE, version 39lift37, Oct 2021).

### Differential gene expression analysis

The R package DESeq2 [10] was then used to identify the DEGs among the PDR and control groups in each RNA-Seq dataset. The cutoff criteria for determining DEGs were |log2 fold change (FC)| > 1 and FDR < 0.05.

### RRA analysis

The Robust Rank Aggregation (RRA) method [11] was used to integrate the results of the RNA-seq studies and control batch effects introduced by different sequencing platforms. A lists of up- and down-regulated genes for each RNA-seq dataset were generated from the expression fold changes between PDR and control groups. RobustRankAggreg package in R was used to integrate and rank differentially expressed genes from each dataset, and genes with a score <0.05 were considered significant in the final integrated dataset.

### Functional and pathway enrichment analysis and construction of protein-protein interaction network

Functional enrichment analysis was used to explore the function of the DEGs, including Gene Ontology (GO) functional enrichment analysis and the Kyoto Encyclopaedia of Genes and Genomes (KEGG) pathway analysis [12, 13], performed by the clusterProfiler package in R [14]. String database (version 11.5) [15] was used to construct a Protein-Protein Interaction (PPI) network based on the DEGs, allowing the names and protein-coding genes of all proteins interacting in the network to be extracted.

### Processing of single-cell RNA-seq data

The scRNA-seq data was processed by the Seurat package in R. First, Canonical Correlation Analysis (CCA) was used to find Mutual Nearest Neighbours (MNNs) [16]. Cells with more than 2,500 or fewer than 200 gene counts, or with more than 5% mitochondria, were filtered out. The “vst” selection method was used to find variable genes, which were input features for initial Principal Component Analysis (PCA) [17]. Jackstraw analysis was then performed to select the Principal Components (PCs) with P-values < 0.05 [18]. Significant PCs were incorporated into further t-distributed Stochastic Neighbour Embedding (t-SNE) to identify different cell clusters with DEGs (resolution = 0.5). The distribution and expression of the top 10 DEGs were displayed on feature plots and heat maps, respectively, while the Blueprint and Encode databases [19–21] in the R package singleR were used as references for defining each cell cluster.

### Identification of the significant cellular communication

The R package CellChat [22] was used to identify ligand-receptor interactions among FVM cells. Venn diagrams were used to illustrate the communication of proteins in the ligand-receptor interactions and PPI networks. We then constructed a PPI network and performed undirected network analysis using cytoscape to identify hub genes. The DisGeNET and DISEASES database was used to identify genes associated with PDR.

### Identification of the key regulons

SCENIC (single-cell regulatory network inference and clustering) method [23] was used to build the regulatory network among FVM cells. We used R packages GENIE3 and RcisTarget to infer the co-expression network and the transcription factor binding motifs. AUCell package was used to identify the active regulons in each cell types.

### GSVA and co-expression analysis

We used GSVA for pathway enrichment analysis of scRNA-seq data. Limma package in R was used to screen pathways with significant differences in different cells. Finally, we verified the correlation between genes and between genes and pathways by the co-expression Pearson correlation analysis of active regulatory genes, key cellular communication genes and differential pathways.

### Validation study

Further validation studies were then conducted using microarray data (GSE60436) on six PDR and three control patients. The Limma algorithm [24] was used to identify DEGs, and genes with the |log2 fold change (FC)| > 1 and P-value <0.05 were considered significant.

## Result

### Differential gene expression analysis

**Fig 1:**
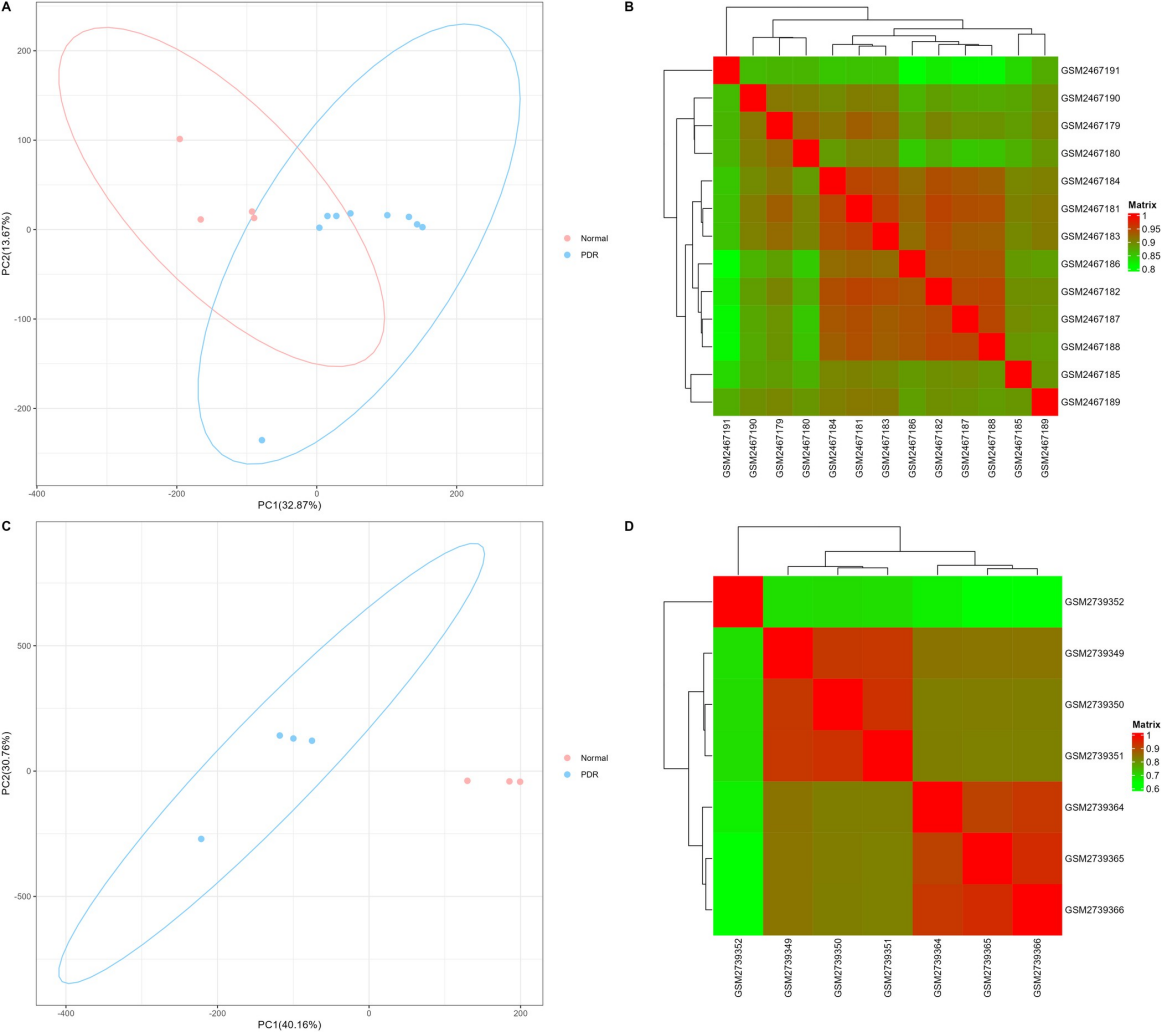
PCA clustering diagram and correlation heatmap of gene expression levels in RNA-seq data. The x-label represents the variance contribution rate of principal component 1, and the y-label represents the variance contribution rate of principal component 2. The color of the heatmap indicates the correlation of gene expression levels in the sample, green represents low correlation and red represents high correlation. (A)-(B) GSE94019. (C)-(D) GSE102485.

**Fig 2:**
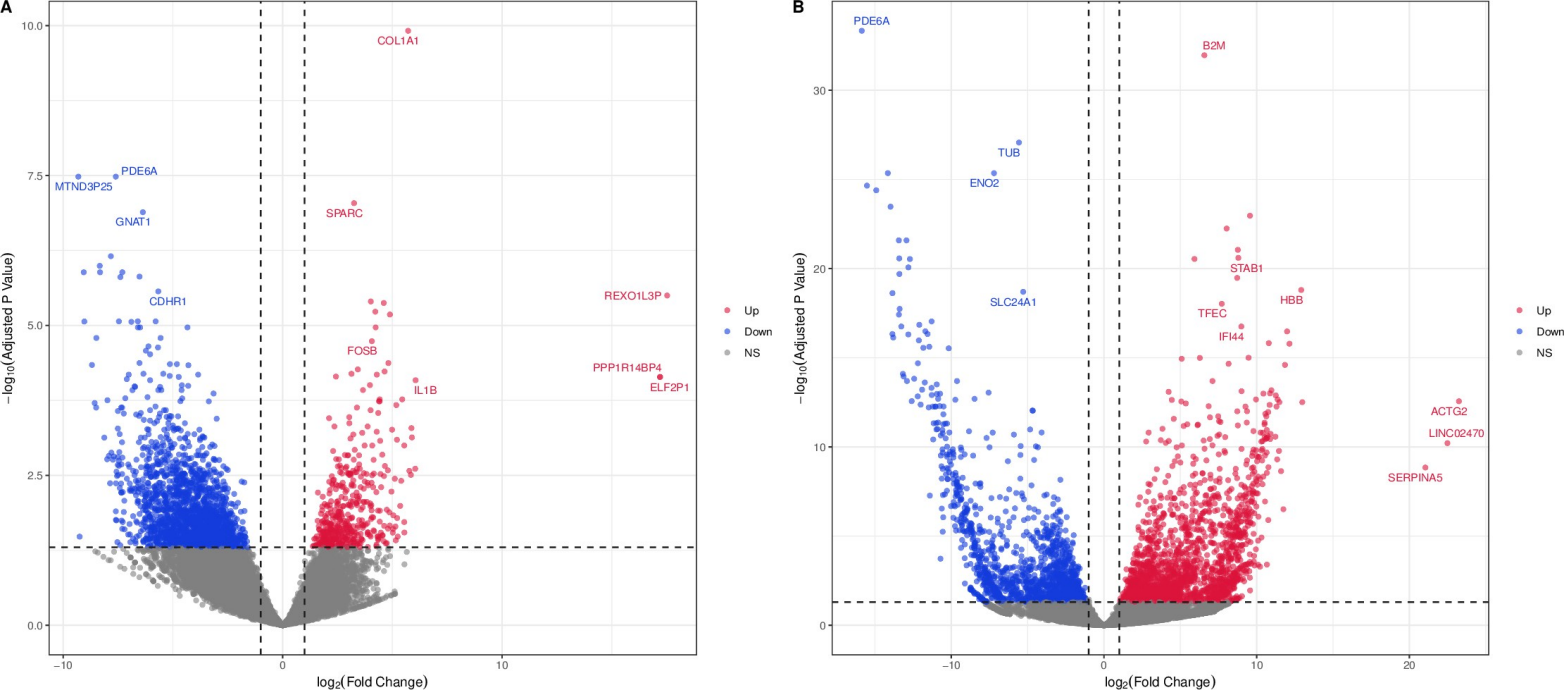
Volcano plots of two RNA-Seq data. Red points represent up-regulated genes and blue points represented down-regulated genes. Gray points represent genes with no significant difference. (A) GSE94019. (B) GSE102485.

The correlation of biological repeat RNA-seq data is shown in Fig 1. All samples were highly correlated (R > 0.6). We first identified DEGs in bulk RNA-seq data according to the cut-off criteria. Volcano plots demonstrating differential expressions are shown in Fig 2A and 2B.

### Results in the RRA integrated analysis

**Fig 3:**
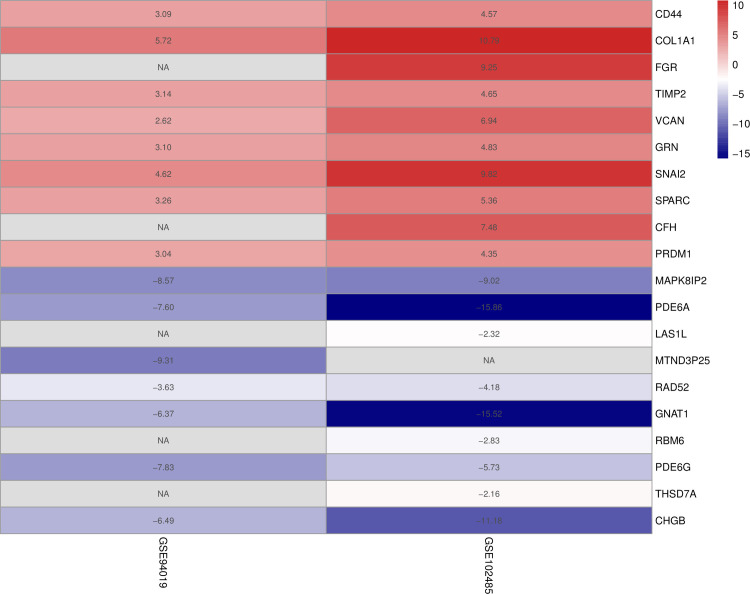
The heatmap of the top 10 up-regulated and down-regulated genes in the RRA analysis. Red indicates high expression of genes in patients with PDR and blue indicates low expression.

Through Robust Rank Aggregation, 176 significant DEGs (81 up-regulated and 95 down-regulated) were identified. The heatmap of the top 10 up-regulated and down-regulated genes is shown in Fig 3.

### Functional enrichment analysis and PPI network analysis

**Fig 4:**
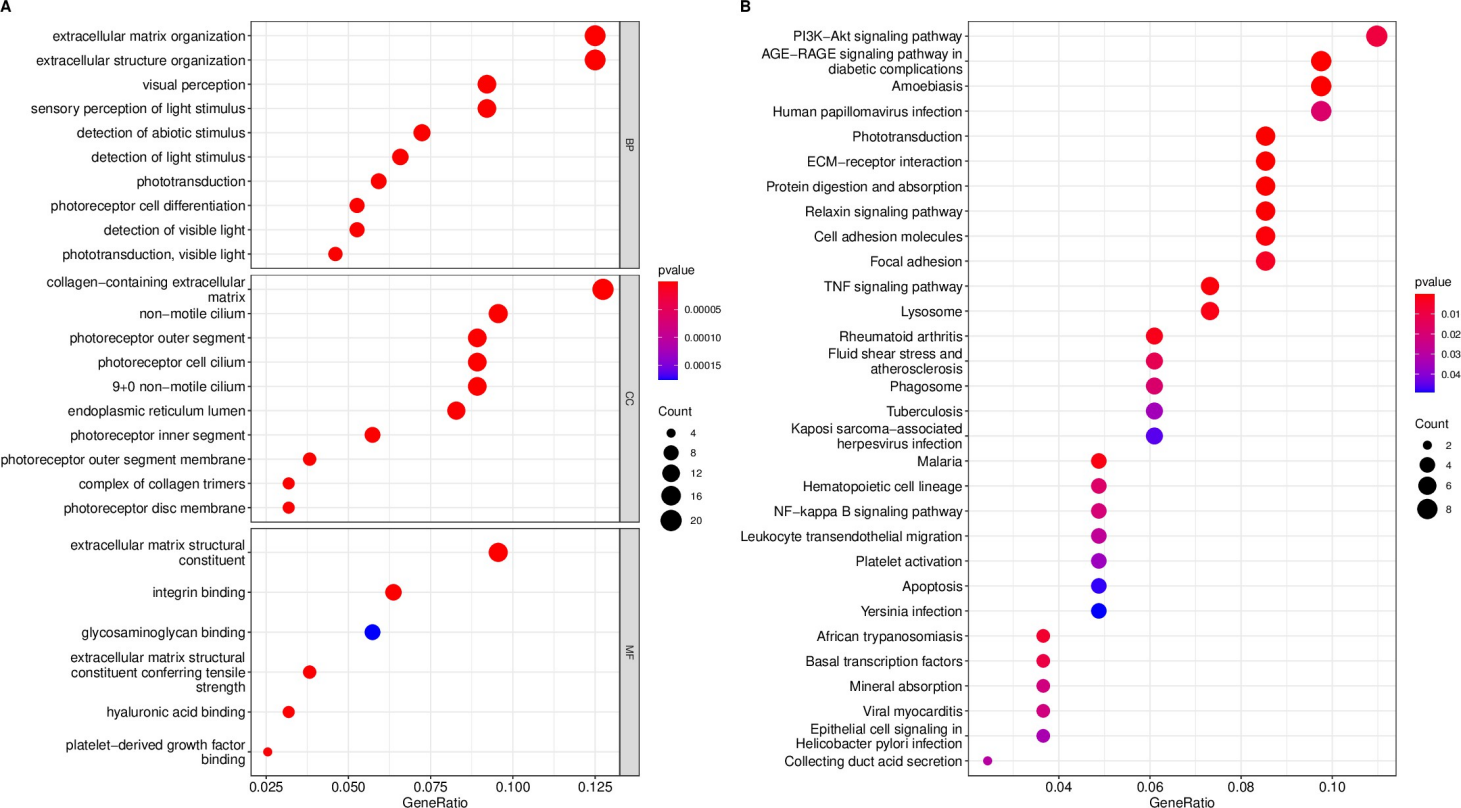
The functional enrichment analysis for DEGs in GO terms and KEGG pathways. (A) The bubble plot of top 10 significant GO terms in biological process (BP), cellular component (CC) and molecular function (MF). (B) The bubble plot of top 30 significant KEGG pathways.

The 176 DEGs were used to perform GO and KEGG analyses. Functional enrichment analysis for these DEGs in GO terms and KEGG pathways are shown in Fig 4A and 4B. Results showed that extracellular matrix structural constituent process (GO:0005201, P-value = 1.67E-11) was most significantly enriched for molecular function, followed by the integrin binding process (GO:0005178, P-value = 4.53E-07) and the hyaluronic acid binding process (GO:0005540, P-value = 1.01E-06) (Table 1). These molecular functions are closely related to fibrosis, cell adhesion and migration, suggesting that these DEGs and their expression products may be involved in the formation of FVMs. Top five KEGG enriched pathways are shown in Table 2. STRING database was used to perform the PPI network analysis of the DEGs. All 176 DEGs included 161 proteins and 392 PPI relationships.

**Table 1:** Top five molecular functions in GO analysis.

ID	Description	p-value	Gene Count
GO:0005201	extracellular matrix structural constituent	1.67E-11	15
GO:0005178	integrin binding	4.53E-07	10
GO:0005540	hyaluronic acid binding	1.01E-06	5
GO:0030020	extracellular matrix structural constituent conferring tensile strength	1.25E-06	6
GO:0048407	platelet-derived growth factor binding	1.62E-06	4

**Table 2:** Top five KEGG pathway enrichment analysis.

ID	Description	p-value	Gene Count
hsa04744	Phototransduction	1.05E-08	7
hsa04933	AGE-RAGE signaling pathway in diabetic complications	6.56E-06	8
hsa05146	Amoebiasis	7.60E-06	8
hsa04512	ECM-receptor interaction	2.66E-05	7
hsa04974	Protein digestion and absorption	7.36E-05	7

### The gene expression landscapes of FVMs cells

**Fig 5:**
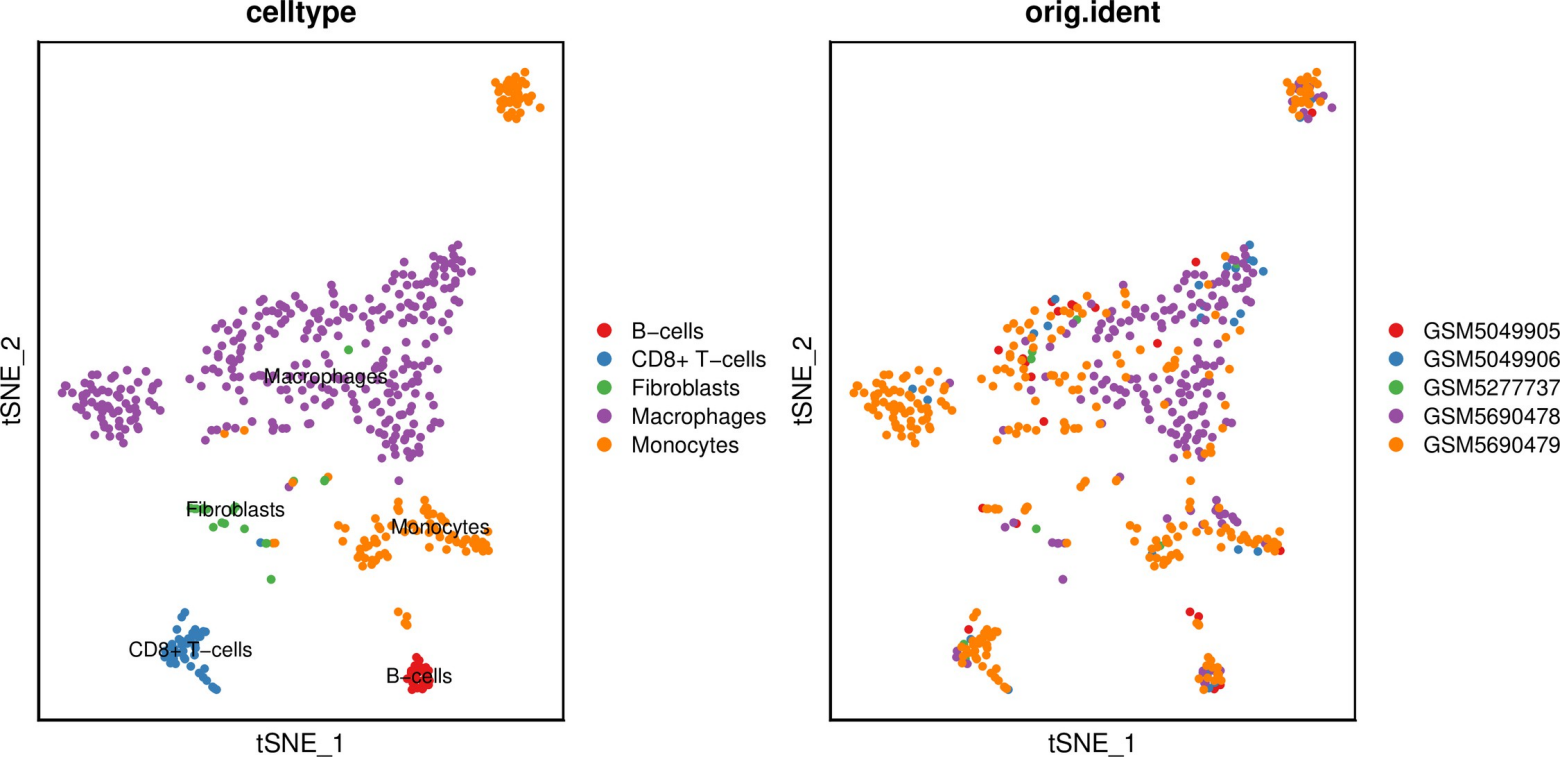
t-distributed stochastic neighbour embedding (t-SNE) plot. Purple represents Macrophages, orange represents Monocytes, red represents B cells, blue represents CD8 + T cells and green represents Fibroblasts.

**Fig 6:**
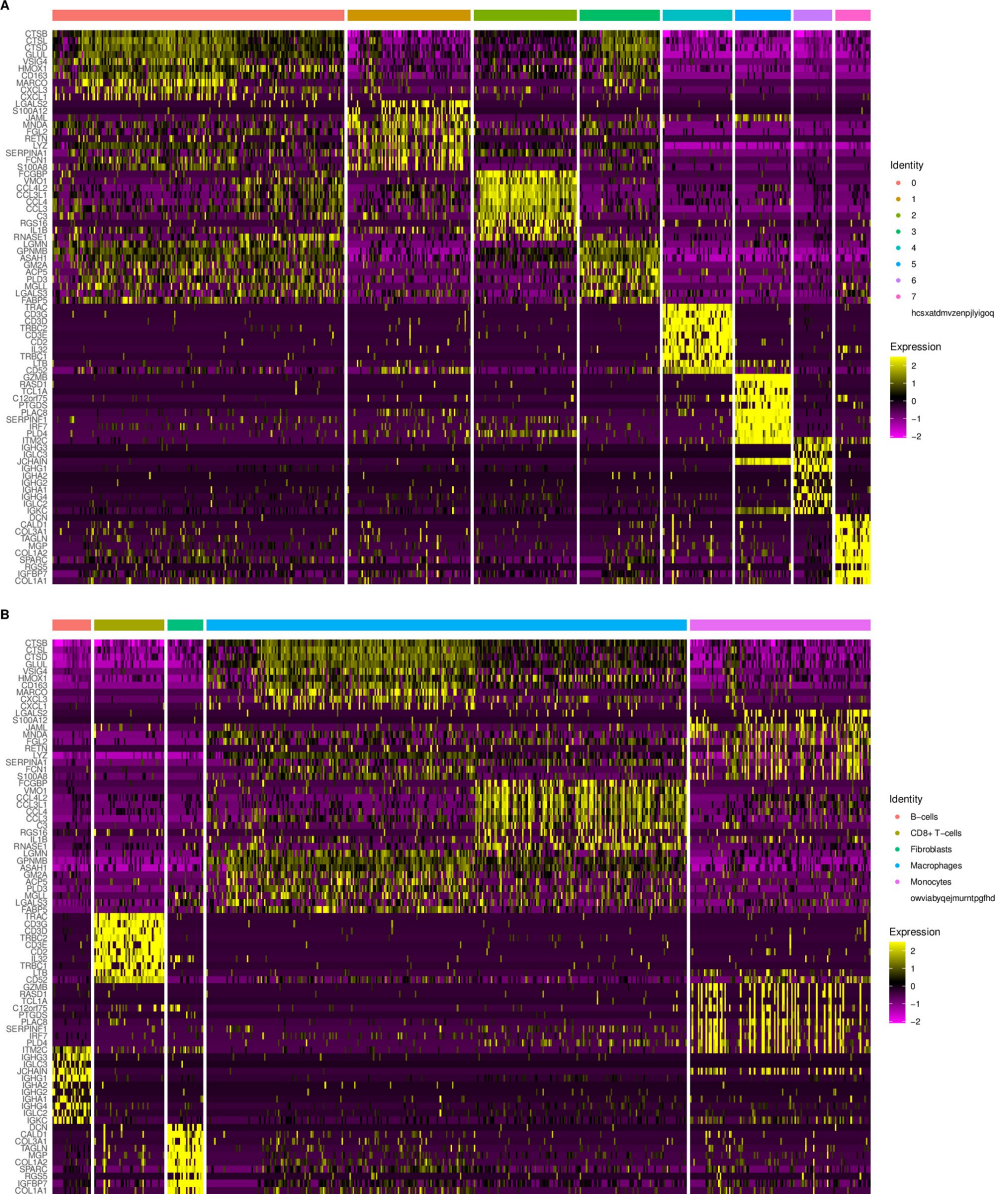
The expression level heatmaps of the top 10 DEGs. (A) The top 10 DEGs in each cluster. (B) The top 10 DEGs in each cell type.

**Fig 7:**
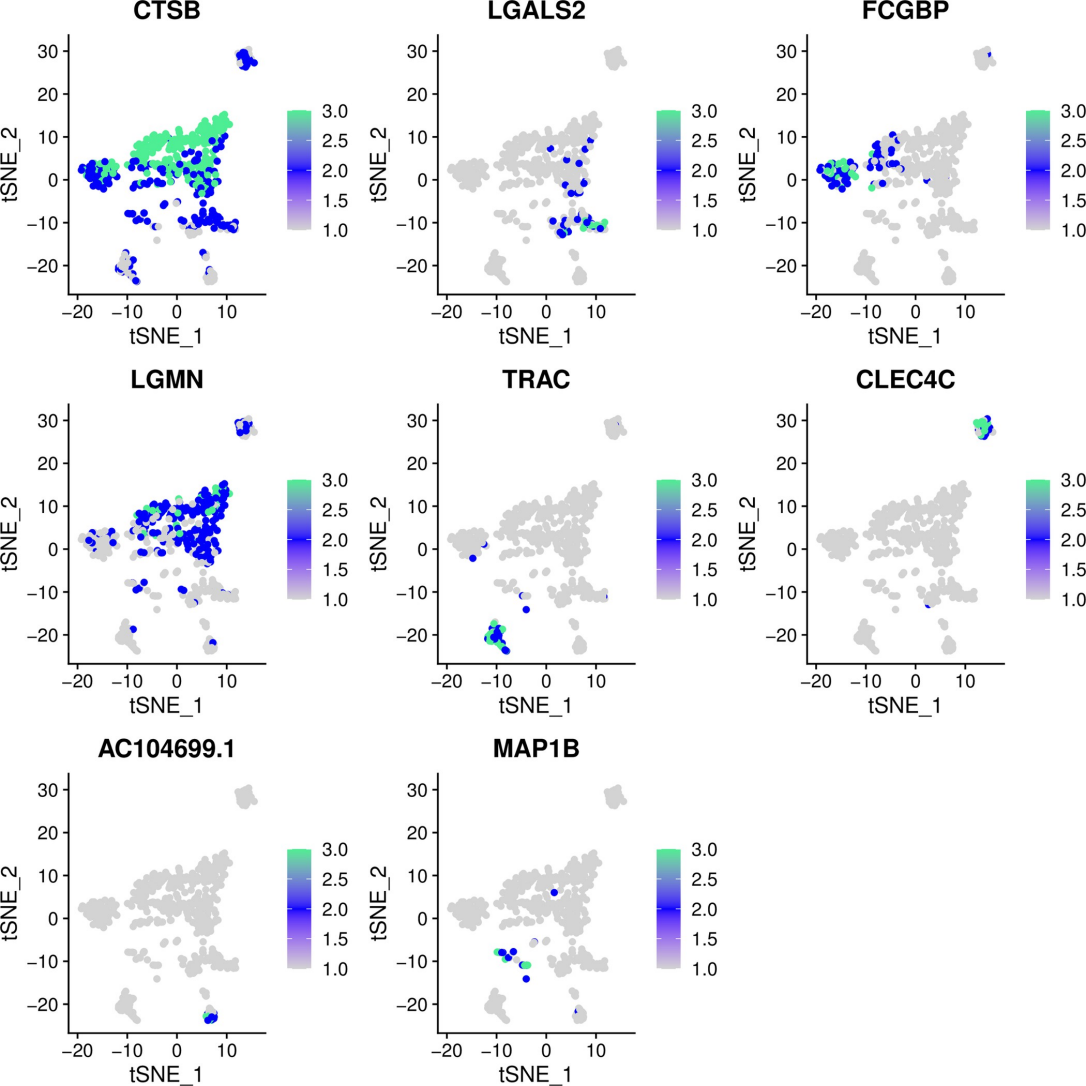
The feature plots of each cell type marker genes. Green represents high expression level and grey represents low expression level.

The t-SNE plot clearly shows eight clusters and five cell types (Macrophages, Monocytes, B cells, CD8 + T cells, Fibroblasts) (Fig 5). Fig 6A and 6B, respectively, show the expression levels of the top 10 DEGs in each cluster and cell type. The feature plots of each cell type marker genes are presented in Fig 7.

### Identification of significant cellular communication in FVMs cells

**Fig 8:**
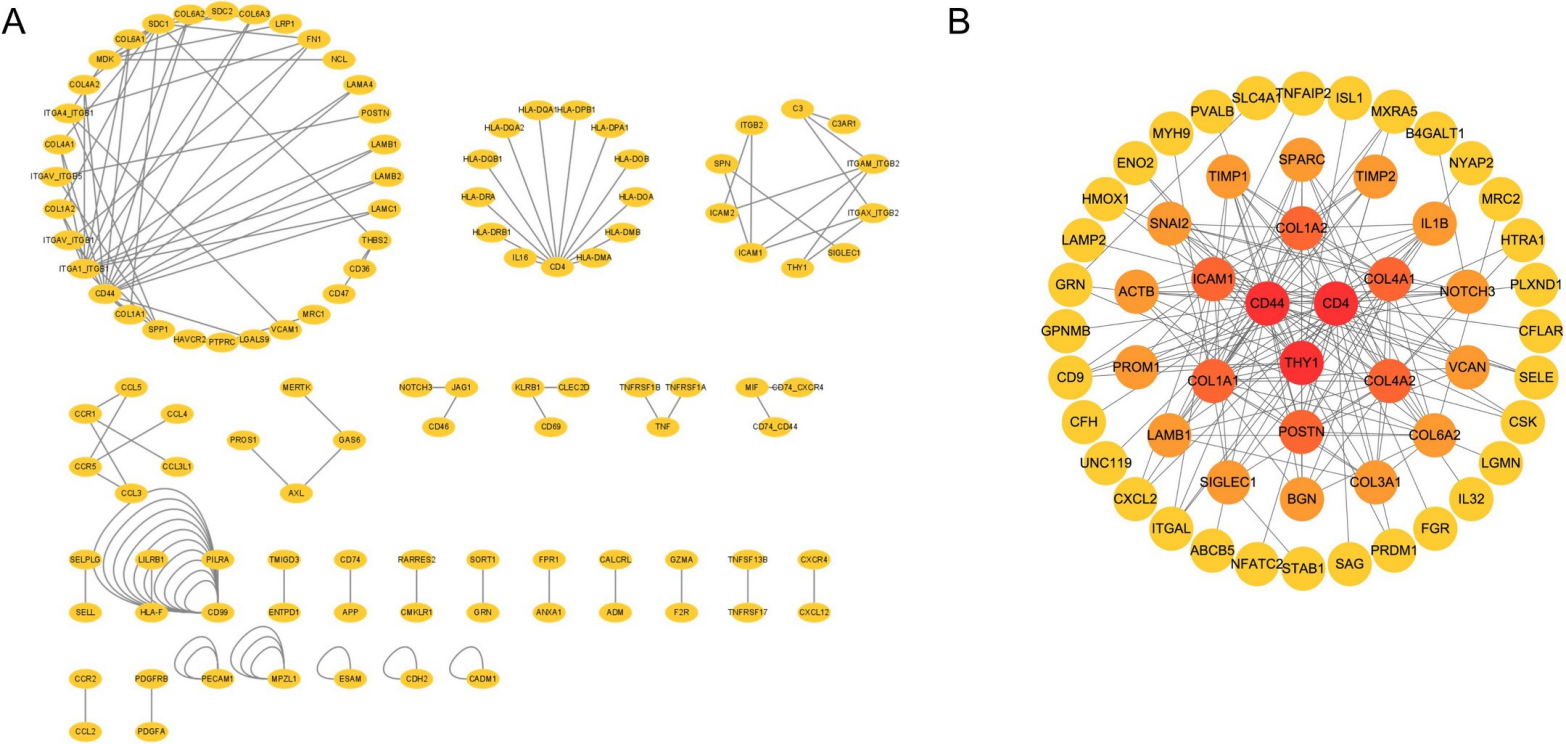
43 ligand-receptor interactions and the PPI network of the interactions between fourteen proteins. (A) 43 significant ligand-receptor interaction networks. (B) PPI network of interactions among CD4, CD44, COL1A1, COL1A2, COL4A1, COL4A2, COL6A2, GRN, ICAM1, LAMB1, NOTCH3, POSTN, SIGLEC1, and THY1. The color represents the degree of protein interactions in the PPI network, with darker color indicating the higher degree.

**Fig 9:**
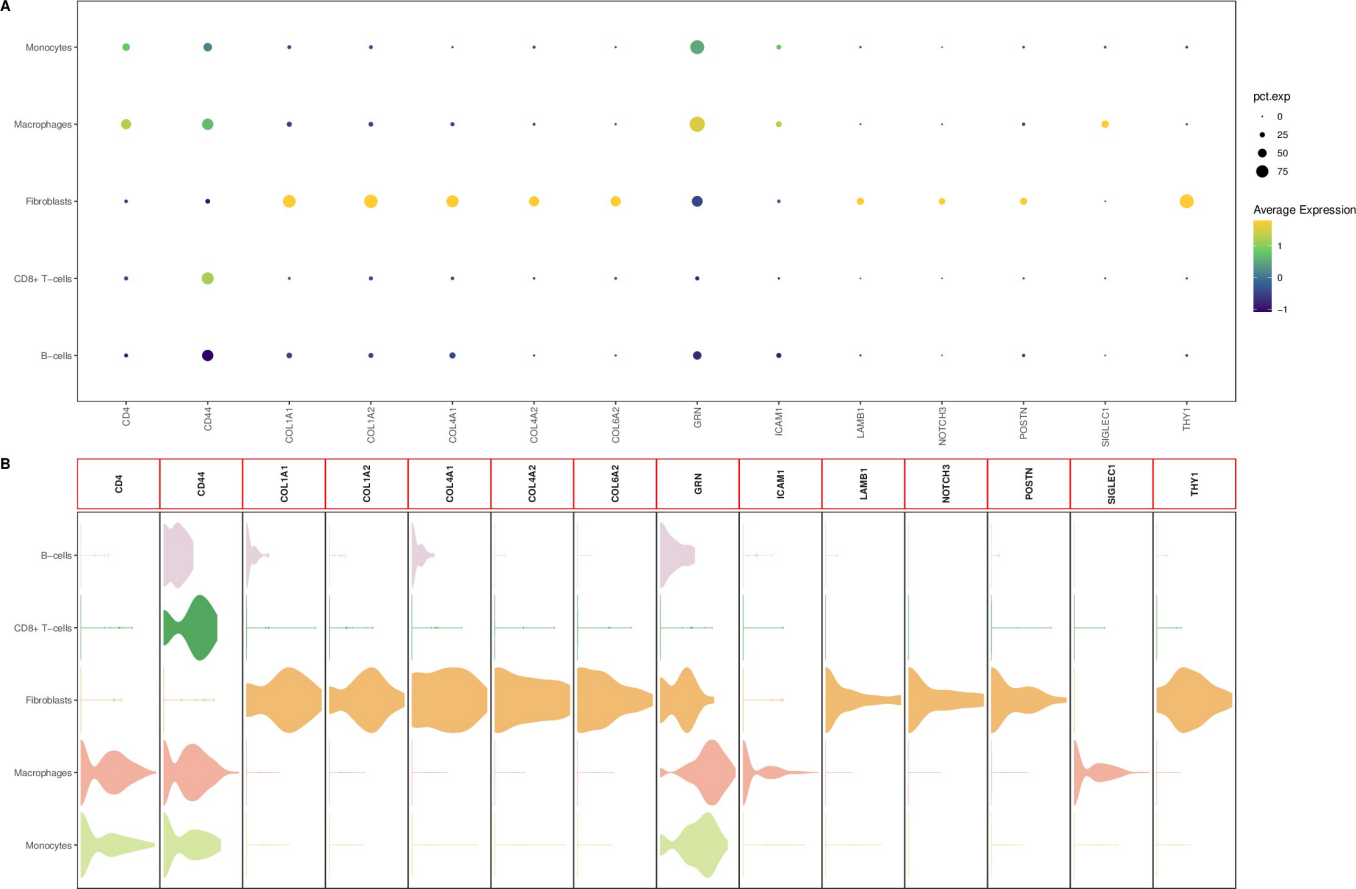
Gene expression levels of CD4, CD44, COL1A1, COL1A2, COL4A1, COL4A2, COL6A2, GRN, ICAM1, LAMB1, NOTCH3, POSTN, SIGLEC1, and THY1 in each cell type. (A) A dot plot shows the expression levels of these genes. The dot size and color represent the percentage of cells expressing the gene and p-values. (B) The violin plot shows the expression distribution of these genes.

**Fig 10:**
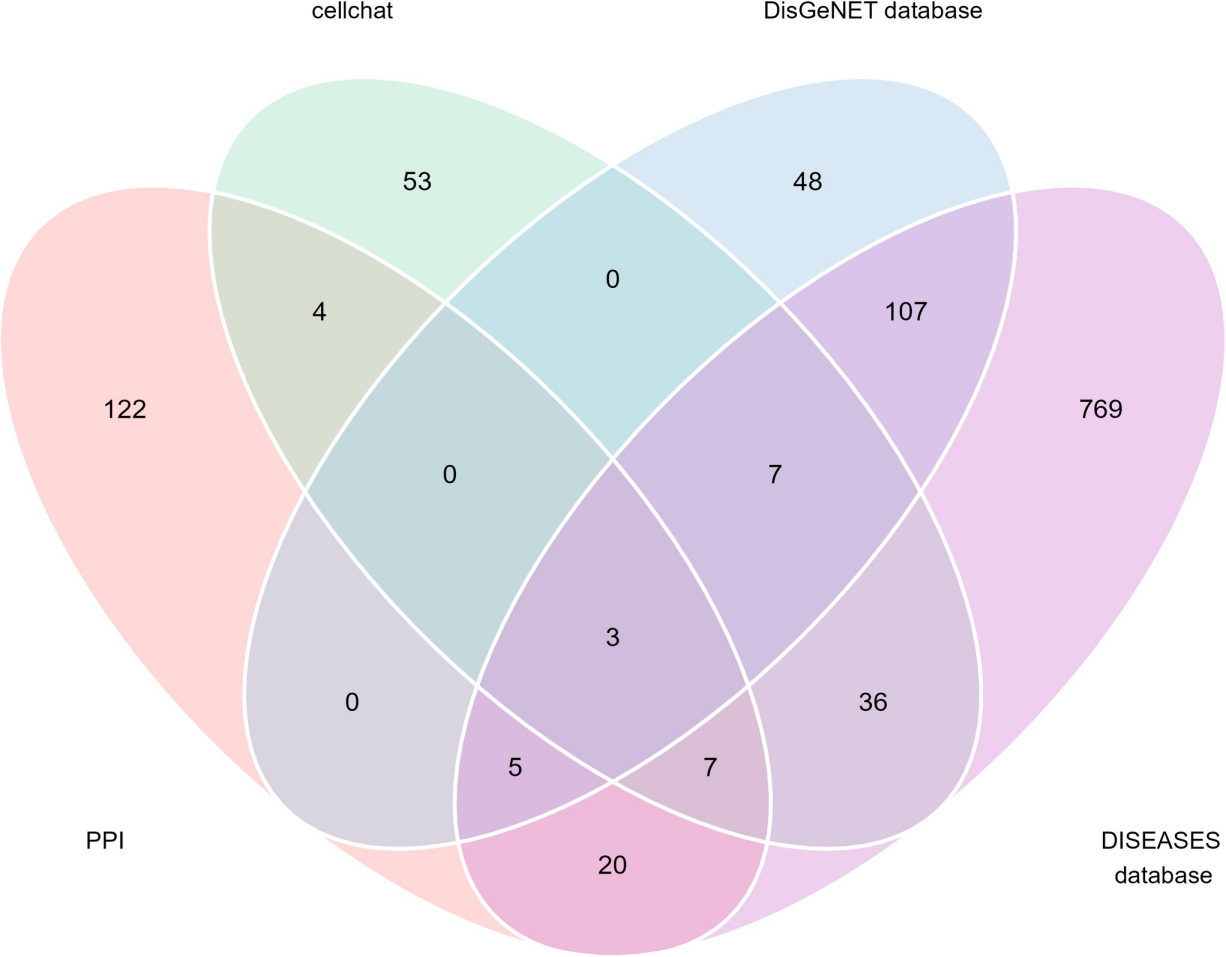
Venn diagram. The Venn diagram shows 14 proteins that not only involved in significant ligand-receptor interactions in FVMs but DEGs-based PPI networks, three of which were associated with PDR.

We then use the CellChat package in R to identify the ligand-receptor interactions among FVMs cells and the DisGeNET database was used to download genes associated with PDR. A total of 43 significant ligand-receptor interactions related to 110 proteins were identified. In addition, 14 proteins (CD4, CD44, COL1A1, COL1A2, COL4A1, COL4A2, COL6A2, GRN, ICAM1, LAMB1, NOTCH3, POSTN, SIGLEC1, THY1) were identified in the overlap of the DEGs-based PPI network and proteins of significant ligand-receptor interactions of FVMs cells. Fig 8A and 8B show 43 ligand-receptor interactions and a new PPI network of the interactions between 14 proteins, respectively. Fig 9A and 9B show the gene expression levels of these 14 proteins in each cell type, respectively. The results show that the coding genes of these 14 proteins are mainly expressed in fibroblasts. We selected the proteins with the top 10 degree in undirected network analysis as hub genes (CD44, CD4, THY1, COL1A1, COL1A2, POSTN, ICAM1, COL4A1, COL4A2, COL6A2), among these proteins, only three (CD44, ICAM1, POSTN) were associated with PDR pathogenesis (Fig 10). Fibroblast-derived POSTN binds to the ITGAV_ITGB5 receptor on fibroblasts through secreted signaling. This interaction may promote fibroblast migration and proliferation. CD44 is involved in cellular interaction between CD8+ T-cells and Macrophages through secreted signaling. These interactions may lead to a direct or indirect control of cellular activities such as adhesion, migration, and proliferation. ICAM1 is mainly involved in cellular interaction between CD8+ T-cells and Macrophages through cell-cell contact, which may mediate cell adhesion and play a critical role in a wide range of biological processes including immune response and inflammation. Altogether, these observations suggest that POSTN, CD44 and ICAM1 may play a key role in the formation of FVMs. Results of CellChat analysis, including POSTN, CD44 and ICAM1, are presented in Table 3.

**Table 3:** The results of cellchat analysis including CD44, ICAM1 and POSTN.

Source	Target	Ligand	Receptor	Prob	Pathway name	Annotation
Fibroblasts	Fibroblasts	POSTN	ITGAV_ITGB5	8.61E-06	PERIOSTIN	Secreted Signaling
Macrophages	CD8+ T-cells	SPP1	CD44	0.0082952	SPP1	Secreted Signaling
	Macrophages			0.0467258		
Macrophages	CD8+ T-cells	LGALS9	CD44	0.0022465	GALECTIN	Secreted Signaling
	Macrophages			0.0123457		
Macrophages	Macrophages	ICAM1	ITGAX_ITGB2	0.0022029	ICAM	Cell-Cell Contact
			ITGAM_ITGB2	0.0020691		
	CD8+ T-cells		SPN	0.0001336		
Macrophages	Macrophages	ITGB2	ICAM1	0.0058955	ITGB2	Cell-Cell Contact

### Identification of the key regulons

**Fig 11:**
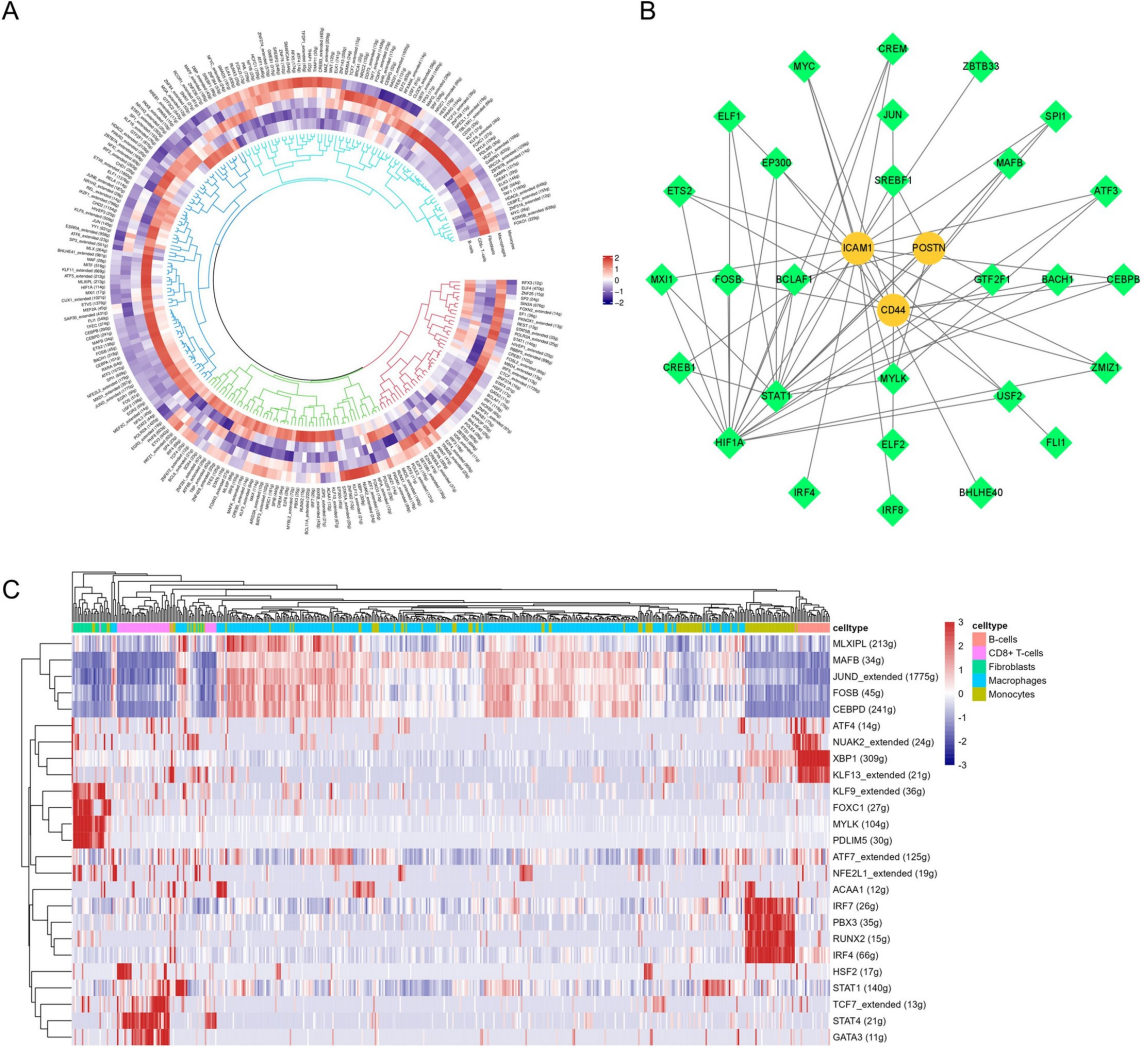
The heatmaps and regulation network of regulators. (A) The heatmap of active regulators in each cell types. Red represents high regulatory activity and blue represents low activity. (B) The regulation network among 28 regulons and 3 key cellular communication genes. Diamond represents regulons and circle represents key cellular communication genes. (C) The heatmap of top five regulons in each cell types. Red represents high regulatory activity and blue represents low regulatory activity.

Through SCENIC analysis, 243 active regulons were identified, 28 of which are associated with POSTN, CD44 and ICAM1. MYLK was one of the top five regulons in fibroblasts (Fig 11A–11C). Therefore, we inferred that MYLK and POSTN are key factors involved in the regulation of fibroblasts.

### GSVA and co-expression analysis

**Fig 12:**
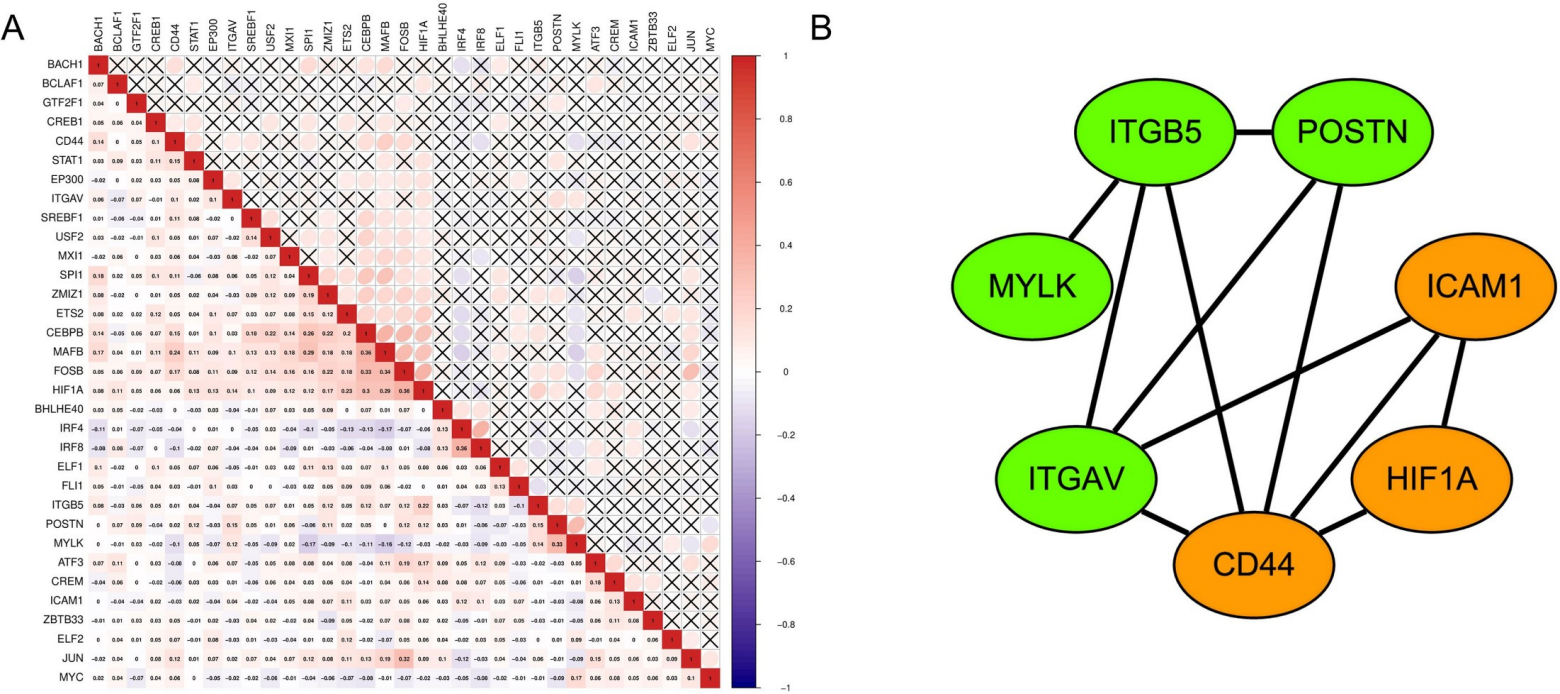
Gene-gene co-expression heatmaps and protein interaction network. (A) The co-expression heatmaps showing R values (the lower triangle) and P-values (the upper triangle), X targets means P-value > 0.05. Red represents positive correlation and blue represents negative correlation. (B) The MCL cluster analysis showing that POSTN, MYLK, ITGVA and ITGB5 are grouped into the same cluster, while CD44, ICAM1 and HIF1A are grouped into another cluster.

**Fig 13:**
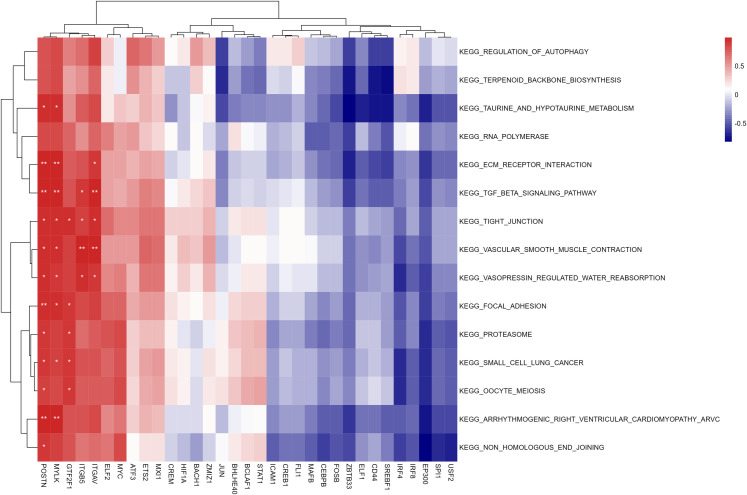
Gene-pathway co-expression heatmap. Red represents positive correlation and blue represents negative correlation. * Indicates p-value < 0.05, ** indicates p-value < 0.01.

We selected the differential pathways in fibroblasts for correlation analysis, showing the co-expression patterns of the active regulons and key cellular communication genes, and their correlation with differential pathways. In co-expression analysis, the regulon MYLK had significantly co-expression pattern with POSTN (R = 0.33, P-value < 0.001). Besides, the regulon HIF1A was co-expressed with ITGVA and ITGB5 (R = 0.14 and R = 0.22, P-value < 0.001), which were the receptors of POSTN and also co-expressed with POSTN (R = 0.12, P-value < 0.001) (Fig 12A). Then we used STRING database to perform MCL (Markov Chains) cluster analysis of these key proteins, and found that the interaction network of key proteins, POSTN, MYLK, ITGAV and ITGB5 are in the same cluster, which is similar to the previous results (Fig 12B). In gene-pathway correlation analysis, POSTN, MYLK, ITGVA and ITGB5 were all significantly associated with pathways related to cell adhesion and migration (P-values < 0.05) (Fig 13). Overall, our study suggested that MYLK, HIF1A and POSTN maybe involved in the proliferation and migration of fibroblasts.

### The validation of study

**Fig 14:**
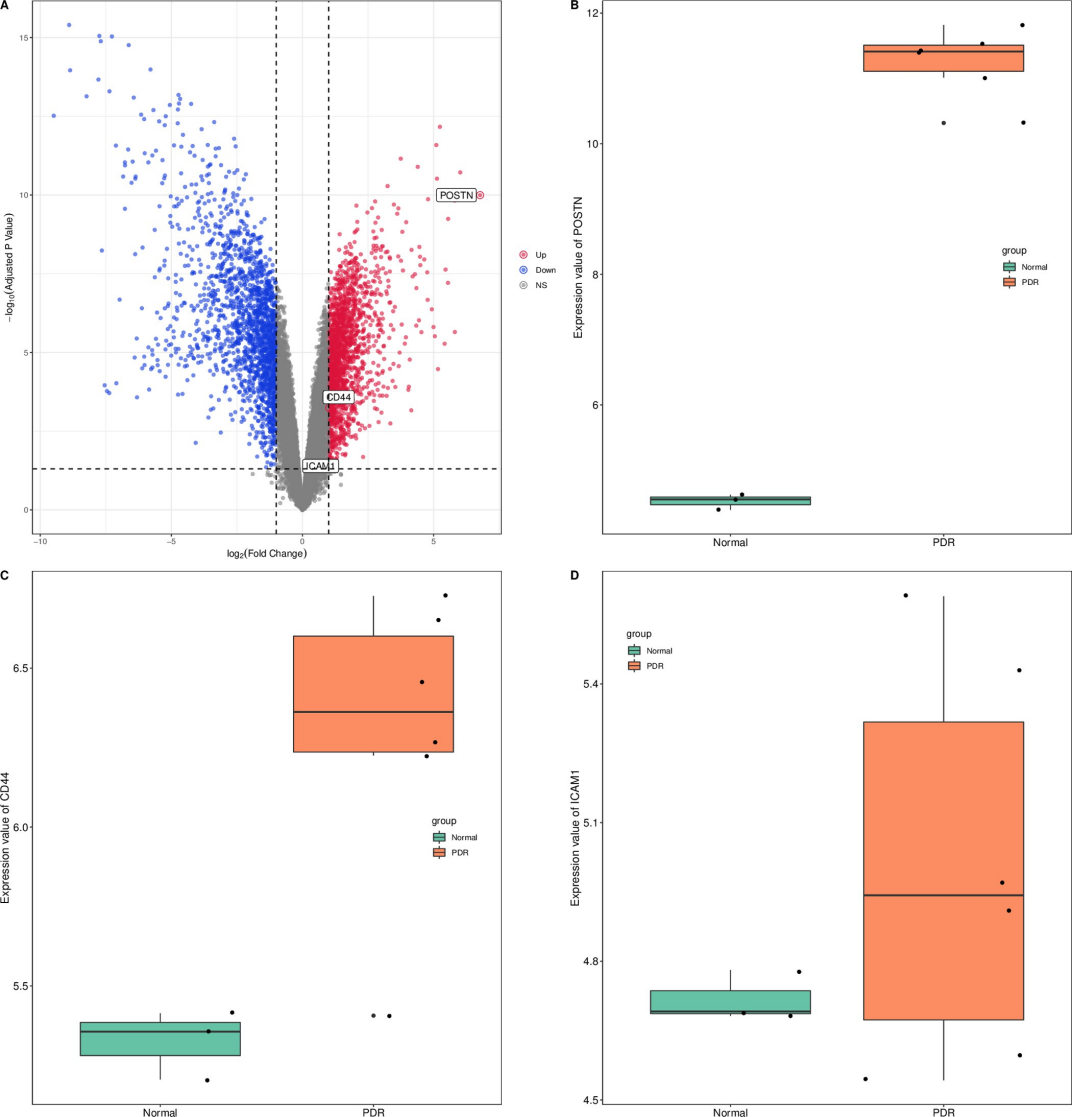
Results of validation using the GSE60436 dataset. (A) A volcan plot showing POSTN and CD44 are significantly upregulated, but ICAM1 is not significantly changed. Red points represented up-regulated genes and blue points represented down-regulated genes, grey points represented genes with no significant difference. (B-D) Boxplots showing that POSTN and CD44 significantly upregulated, no significant change in ICAM1.

Finally, we validated our results by using microarray data GSE60436, and found that POSTN (P = 1.01^−10^) and CD44 (P = 5.78^−4^) had significant differences in the verification dataset. Only ICAM1 showed no significant difference in the validation dataset (P = 0.09). Verification results are presented in Fig 14A–14D.

## Discussion

PDR is a common chronic complication in diabetic patients, and progression of the disease may eventually result in irreversible visual impairment, underscoring the critical importance of its early diagnosis and treatment. Current PDR treatments include control of metabolic disorders, anti-VEGF therapy, laser therapy and surgery, but all have limitations such as multiple drug injections, vitreous surgeries and poor prognosis. Through transcriptomic and pathway analysis, our findings provide new targets and directions for PDR’s early intervention and treatment.

As PDR develops, the molecular and cellular characteristics of the disease often change, making it possible to screen out important predictors; thus, DEGs and cell communication in FVM cells are of interest to this research. This study is the first to perform integrated transcriptomic analyses based on bulk RNA and single-cell RNA sequencing, using two bulk RNA-seq datasets and one single-cell RNA-seq dataset to compare gene expression profiles between PDR and control patients and RRA analysis to give results greater statistical power. In addition, functional annotation and PPI network construction were performed to understand the potential biological functions of DEGs.

From a total of 176 DEGs and 43 ligand-receptor pathways, 14 proteins involved in both the DEG-based PPI network and significant ligand-receptor interactions in FVM cells. Three of them were associated with PDR (POSTN, ICAM1, CD44). By SCENIC analysis, we identified POSTN as the key ligand.

Periostin (POSTN) is a Fasciclin family stromal cell protein [25] that is believed to initiate cell proliferation, migration and epithelial-to-mesenchymal transformation by interacting with several integrins, including αVβ3, αVβ5 and α6β4 [26, 27]. Our study found POSTN to be significantly expressed in fibroblasts. Fibrosis is a recognised marker of advanced PDR, with studies showing significantly more active fibroblasts in PDR FVMs than in idiopathic epiretinal membranes, suggesting that fibroblasts are crucial to PDR development [28]. POSTN in fibroblasts is thought to be involved in many fibrotic diseases through TGF-β (Transforming Growth Factor -β) and/or TNFα/IL-1α (Tumour Necrosis Factor α/ Interleukin -1α) interactions [29]. POSTN is also believed to act directly on fibroblasts, leading to their activation and proliferation. One study suggests that POSTN promotes idiopathic pulmonary fibrosis through mesenchymal effects, cross-linking collagen and stiffening the matrix created by fibroblasts, thereby activating cells for further production of the extracellular matrix [30]. POSTN directly activates mesenchymal cells, or induces expression of TGF-β from these cells, to accelerate the inflammation and fibrosis process [29, 30]. Additionally, POSTN is thought to be involved in cell proliferation and migration by binding to integrins αVβ3 or αVβ5 and activating FAK/Akt phosphorylation [31], this has also been proved in our research. In the present study, fibroblast-derived POSTN acted as a ligand for fibroblasts’ integrin αVβ5 receptors by secreting signals, suggesting there may be a POSTN-dependent autocrine effect in fibroblast proliferation. Besides, studies have showed that POSTN is involved in vascular remodelling in a variety of diseases [32–34]. These processes may be important for promoting fibroblast involvement in FVM formation in diabetic retinas, so inhibiting POSTN may contribute to PDR treatment.

In our study, we found that MYLK and HIF1A are involved in the regulation of POSTN. MYLK is known as an important marker of fibrosis [35, 36]. POSTN has been reported to activate integrin signaling, which initiates MYLK phosphorylation and leads to reorganization of the actin cytoskeleton important for the regulation of vascular permeability and cell migration [37]. This activation of integrin signaling may also lead to increased HIF1A expression by initiating PI3K/Akt/mTOR signaling pathway, resulting in increased VEGF and angiogenesis [38, 39], and it has been found that there is a positive feedback loop between HIFs and the POSTN signal [32]. HIF expression increases during tissue hypoxia, which may be the reason for POSTN overexpression in PDR patients.

Traditional treatments for PDR include vitreous surgery and intraocular injection of anti-VEGF drugs, but such treatments are often associated with poor efficacy, multiple surgeries and treatment resistance, meaning that safer, more effective approaches are necessary. RNA interference-based therapeutics targeting key genes provides one possible solution. A single-stranded RNAi agent targeting POSTN has demonstrated inhibiting behaviours to Choroidal Neovascularization (CNV) formation with good stability and no serious toxicity [40]. In an in-vitro trial, the POSTN-targeted inhibitor also demonstrated effectiveness in inhibiting retinal neovascularization [31]. These findings indicate that POSTN is a good prospect as a new therapeutic target for PDR, with POSTN inhibitors expected to become new therapeutic agents for PDR in the future.

ICAM1, or Intercellular Adhesion Molecule 1, is the ligand for the leukocyte adhesion protein LFA-1 (integrin alpha-L/beta-2). During leukocyte trans-endothelial migration, ICAM1 engagement promotes the assembly of endothelial apical cups through ARHGEF26/SGEF and RHOG activation, binding cells together or to the extracellular matrix. The molecule contributes to cell proliferation, differentiation, motility, trafficking, apoptosis and tissue architecture. Rangasamy et al. [41] suggest that hyperglycemia leads to increased ICAM1 expression in retinal microvascular endothelial cells, activating leukocytes and resulting in their attachment to endothelial cells. Leukocyte attachment leads to microvascular damage through the secretion of pro-inflammatory molecules (VEGF, TNFα, Ang-2, proteinases, chemokines) and leukocyte deposition. In addition, several studies document elevated ICAM1 levels in PDR patients’ vitreous fluids [42–44].

The CD44 antigen is the receptor for Hyaluronic Acid (HA), mediating cell-cell and cell-matrix interactions through affinity for HA and possibly through affinity for other ligands such as osteopontin, collagens and Matrix Metalloproteinases (MMPs). CD44 is believed to be involved in tumour growth and metastasis, proliferative diabetic retinopathy and atherosclerosis [45]. In a recent study, AGEs (Advanced Glycation Endproducts) were found to interact with CD44 to form stress fibres and RMP (Retinal Microvascular Pericytes) migration, causing pericytes to detach from microvessels and damage vascular integrity [46]. Moreover, activation of the CD44 receptor signalling pathway may result in the release of multiple inflammatory factors, while up-regulation of ICAM-1 indirectly leads to increased endothelial cell activation [45].

In summary, this study used the Robust Rank Aggregation to integrate multiple bulk RNA-seq datasets, and combing single-cell expression information to provide deeper insight into the comprehensive molecular changes of PDR pathogenesis.

Still, several limitations of this study including the scarcity of public data and heterogeneity introduced by integrating different batches of experiments should be considered. While the RRA method can reduce these differences, bias may still exist. Future studies should collect more tissues from PDR patients and controls to identify additional PDR markers and therapeutic targets.

## Conclusion

By overlapping the DEGs with from PDR-associated gene list from DisGeNET and DISEASES database, we identified the upregulation of POSTN, ICAM and CD44 as biomarkers of the disease. Among them, POSTN activates FAK/Akt phosphorylation by binding with integrin αvβ5 on fibroblasts through autocrine, leading to the proliferation and migration of fibroblasts and the formation of FVMs. This pathway may be considered as potential therapeutic targets for PDR treatment.
